# Non-active Site Residue in Loop L4 Alters Substrate Capture and Product Release in d-Arginine Dehydrogenase

**DOI:** 10.1021/acs.biochem.2c00697

**Published:** 2023-02-16

**Authors:** Daniel Ouedraogo, Michael Souffrant, Xin-Qiu Yao, Donald Hamelberg, Giovanni Gadda

**Affiliations:** † Department of Chemistry, Georgia State University, Atlanta, Georgia 30302, United States; ‡ Department of Biology, Georgia State University, Atlanta, Georgia 30302, United States; § Center for Diagnostics and Therapeutics, Georgia State University, Atlanta, Georgia 30302, United States; ∥ Center for Biotechnology and Drug Design, Georgia State University, Atlanta, Georgia 30302, United States

## Abstract

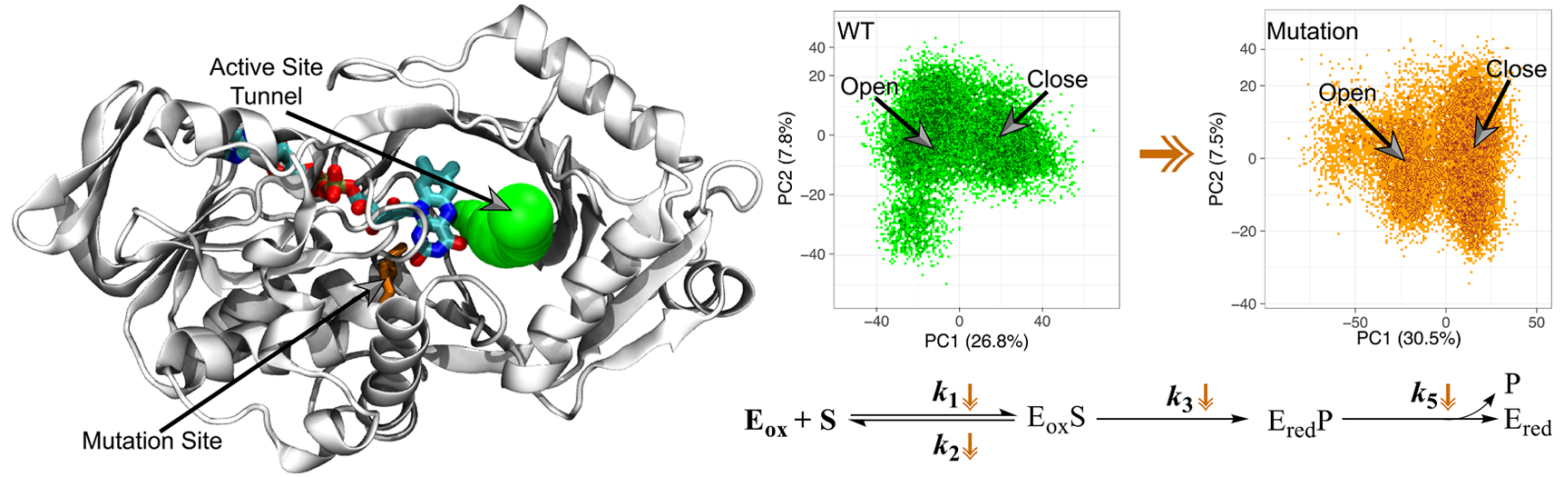

Numerous studies demonstrate that enzymes undergo multiple conformational changes during catalysis. The malleability of enzymes forms the basis for allosteric regulation: residues located far from the active site can exert long-range dynamical effects on the active site residues to modulate catalysis. The structure of *Pseudomonas aeruginosa*d-arginine dehydrogenase (*Pa*DADH) shows four loops (L1, L2, L3, and L4) that span the substrate and the FAD-binding domains. Loop L4 comprises residues 329–336, spanning over the flavin cofactor. The I335 residue on loop L4 is ∼10 Å away from the active site and ∼3.8 Å from N(1)–C(2)=O atoms of the flavin. In this study, we used molecular dynamics and biochemical techniques to investigate the effect of the mutation of I335 to histidine on the catalytic function of *Pa*DADH. Molecular dynamics showed that the conformational dynamics of *Pa*DADH are shifted to a more closed conformation in the I335H variant. In agreement with an enzyme that samples more in a closed conformation, the kinetic data of the I335H variant showed a 40-fold decrease in the rate constant of substrate association (*k*_1_), a 340-fold reduction in the rate constant of substrate dissociation from the enzyme–substrate complex (*k*_2_), and a 24-fold decrease in the rate constant of product release (*k*_5_), compared to that of the wild-type. Surprisingly, the kinetic data are consistent with the mutation having a negligible effect on the reactivity of the flavin. Altogether, the data indicate that the residue at position 335 has a long-range dynamical effect on the catalytic function in *Pa*DADH.

## Introduction

Enzymes are dynamic molecules that operate as biocatalysts with high efficiency to sustain life. Since the 1960s, X-ray crystallography has provided insights into enzyme function.^1,2^ X-ray protein structures reveal the identity and the extensive networks of amino acid residues within a protein but have limitations in fully explaining how these networks of residues work synergistically in catalysis.^1,2^ Increasing evidence demonstrates that enzymes do not remain static or work exclusively close to their native-state structure represented by the X-ray crystal structure but undergo multiple conformational changes during catalysis.^3,4^ Typical enzymatic catalysis is a dynamic process that includes substrate binding, a chemical step involving reorganizing active site residues for covalent bond rearrangements and product release.^5,6^ Non-catalytic residues in loops often participate in the dynamical processes required to position other residues relevant for ligand binding and catalysis.^7−10^ Recent studies have indicated that non-active site residues or residues positioned away from the active site also contribute to the rate acceleration of enzymes.^7,11,12^ The correlation of structure–function and dynamics in proteins has been extensively studied for several years; however, a detailed understanding of how conformational dynamics orchestrate catalysis remains a grand challenge.^13−15^

**Figure 1:**
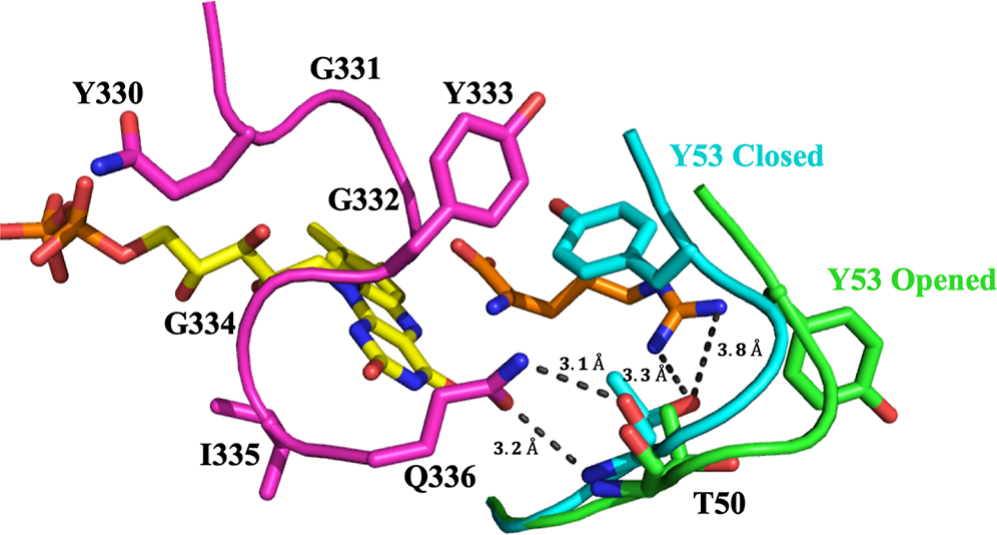
Interaction between loop L4 (magenta) and loop L1 (green/blue) in *Pa*DADH. The FAD cofactor is represented by its isoalloxazine ring with the C atoms in yellow. The opened conformation of loop L1 is shown in green, while the closed conformation is shown in blue. Only the substrate-binding domain of loop L1 is represented.

**Scheme 1:**
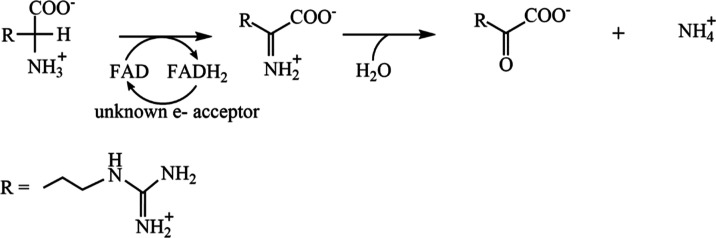
d-Arginine Oxidation by *Pa*DADH

One of the essential factors for understanding the correlation between protein dynamics and catalysis is to probe the synergistic effects of residues in remote sites that have a long-range dynamical impact on the active site residues. Mutation of residues not in direct contact with the ligands could trigger a change in the protein conformation and dynamics, affecting catalysis at the active site. These mutations yield conceptually similar results to binding biological molecules in an allosteric site to induce conformational changes,^16−19^ and we refer to them as allosteric mutations. A compelling case for dynamically coupled long-range effects by remote residues is human cyclophilin A, a peptidyl-prolyl isomerase with an extensive range of biological functions.^20^ Nuclear magnetic resonance and computational studies identified V6 and V29, located ∼15 Å away from the enzyme’s active site, as two “hot spot” residues that were shown to affect catalysis upon mutation.^21,22^ Another example is human monoacylglycerol lipase (hMGL), which belongs to the α/β hydrolase superfamily and uses a nucleophile–histidine–aspartate catalytic triad for catalysis.^23^ W289 and L232, located ∼15 Å from the catalytic triad, were identified as critical residues contributing to the structural integrity of hMGL.^24^ The W289L and the L232G mutations showed a 10^5^-fold loss in catalytic efficiency, indicating that these residues are involved in a residue network that controls signal propagation to the active site and thus regulates the interconversion between the active and inactive states of hMGL.^24^ Moreover, the mutation of G121 to valine in the *Escherichia coli* dihydrofolate reductase (DHFR) FG loop, which is 15 Å away from the active site, perturbs the closed conformation of DHFR, leading to a 40-fold decrease in NADPH binding and a 200-fold reduction in the hydride transfer rate.^11,25,26^ The S148A mutation in the DHFR GH loop, which is also distal to the active site, results in ligand affinities and off-rates changes.^11,25,26^

d-arginine dehydrogenase from *Pseudomonas aeruginosa* PAO1 (*Pa*DADH) is a flavin-dependent enzyme that catalyzes the oxidative deamination of d-amino acids into their corresponding α-keto acids and ammonia (Scheme 1).^27−29^ The physiological role of *Pa*DADH is to catalyze the first step of a coupled-enzyme D- to l-arginine conversion, allowing *P. aeruginosa* PAO1 to subsist with d-arginine as a sole source of carbon and nitrogen.^29^ Most d-amino acids except d-glutamate and d-aspartate are substrates for the enzyme, with d-arginine being the best substrate, displaying the highest *k*_cat_/*K*_m_ value of 3 × 10^6^ M^–1^ s^–1^.^30^ Previous kinetic investigation of the *Pa*DADH wild-type enzyme established the formation of an anionic hydroquinone form of the reduced flavin, which harbored a negative charge close to the N(1)–C(2)=O atoms of the flavin.^31^ In most flavin-dependent enzymes reacting with oxygen, the flavin N(1)–C(2)=O atoms are proximal to a positively charged protein entity,^32^ either provided by the dipole N-terminus of an α-helix, a cluster of peptide nitrogen atoms,^33,34^ or the side chain of a fully charged histidine, lysine, or arginine.^34−40^ A positive charge close to the flavin N(1)–C(2)=O atoms is considered to stabilize the anionic form of the reduced flavin, which is common in oxidases but not in dehydrogenases.^32^ In the crystal structure of *Pa*DADH, the closest protein side chain to the flavin N(1)–C(2)=O atoms is I335, at a distance of ∼3.8 Å (Figure 1), and no positive charges are located nearby.^41^ As shown in Figure 1, loop L4, which harbors I335, interacts with loop L1 through Q336 and T50. Loop L1 was shown to act as an active site lid that can adopt either open or closed conformations, with important implications for substrate capture and catalysis.^42^ Thus, I335 in loop L4 might contribute to the overall conformational dynamics and catalysis of the enzyme.

In the present study, I335 of *Pa*DADH was mutated by site-directed mutagenesis to a small polar side chain residue, histidine, to probe the effect of changing the microenvironment around the flavin N(1)–C(2)=O atoms on the catalytic function of the enzyme. A computational and mechanistic investigation of the I335H variant was carried out, showing that the non-active site I335 in loop 4 alters substrate capture and product release with a minimal role in flavin reduction despite its proximity to the flavin N(1)–C(2)=O atoms, and its location outside the enzyme’s active site would have suggested otherwise.

## Experimental Procedures

### Materials

*E. coli* strain DH5α was obtained from Invitrogen (Grand Island, NY) and Rosetta(DE3)pLysS was obtained from Novagen (Madison, WI). *Pfu* DNA polymerase was obtained from Stratagene (La Jolla, Ca). Deoxy-ribonucleotides were purchased from Sigma Genosys (The Woodlands, TX). The QIA prep Spin Mini-Prep kit and QIA quick polymerase chain reaction (PCR) purification kits were obtained from Qiagen (Valencia, CA). Luria–Bertani agar, Luria–Bertani broth, chloramphenicol, isopropyl β-d-thiogalactonopyranoside, lysozyme, phenazine methosulfate (PMS), and phenylmethanesulfonyl fluoride were purchased from Sigma-Aldrich (St. Louis, MO). Bovine serum albumin was obtained from Promega (Madison, WI). All other reagents were of the highest purity commercially available.

### Molecular Dynamics

The initial structures of the wild-type and the I335H variant were generated from the Protein Data Bank (PDB) entry 3NYC.^43^ The mutation system was created by changing the sequence of the enzyme in the PDB, while removing the wild-type side chain atoms at I335. The LEAP module from AmberTools added the missing side chain atoms for the mutant variant. The systems were sampled through the Amber14 suite of programs^44^ and a modified version of the Amber ff14SB^45^ developed by Cornell et al.^46^ Each system was solvated with a 10 Å TIP3P octahedral box^47,48^ and neutralized with counter ions. The I335H variant was constructed through the LEAP module in AmberTools. The FAD parameters came from the GAFF amber force field.^49^ PROPKA was used to specify the protonation states of histidine residues. The SHAKE algorithm was applied to constrain bonds involving hydrogen atoms.^50^ Both the wild-type and the I335H variant were simulated with a 2 fs time step, at the constant pressure and temperature of 1 bar and 300 K, respectively. A Monte Carlo barostat with a coupling constant of 1.0 ps was used to define pressure. A Langevin thermostat with a collision frequency of 1.0 ps^–1^ was applied to a specified temperature. The particle mesh Ewald (PME) summation method was assessed for long-range electrostatic interactions.^51^ Short-range non-bonded interactions were cut off at 9 Å. Each system was minimized and allowed to equilibrate for 1000 steps and 2.5 ns, respectively. During minimization and equilibration, protein atoms were restrained harmonically at a decreasing force constant from 100 to 0 kcal/mol Å^2^. Each system was sampled for a total of 2.1 μs; the first 100 ns was considered to be equilibration and was removed from subsequent analyses.

Root mean square fluctuation (RMSF) and distance analyses were performed using cpptraj of AmberTools.^52^ The distance analysis studies were carried out by following the same procedure as that used in a previous study with *Pa*DADH.^42^ The flavin cofactor was localized and its fluctuation allowed for the evaluation of the distance analyses between the N5 atom of the flavin cofactor and the C4 atom of Y53. This approach was feasible due to the planarity of the flavin with regard to the N1, N5, and C9 atoms.

Substrate entrance cavities were assessed through CAVER 3.0.^53^ The lowest-cost tunnel from the flavin N5 atom of the FAD to the bulk solvent was selected for every 2000 snapshots of each trajectory. Tunnels were represented as spherical probes. The smallest ball of a tunnel was denoted the tunnel bottleneck. The bottleneck radii over the simulation time frame were generated as a heat map for the wild-type and the I335H variant.

Principal component analysis (PCA) was performed through AmberTools,^52^ where the top two principal components (PC1 and PC2) were plotted using the ggplot2 R package.^54^ PC1 and PC2 eigenvectors were shown through Visual Molecular Dynamics.^55^ Contact statistics were assessed for both systems as shown in Doshi et al.^56^ Contact formation was determined to be optimized at a distance cutoff of 4.5 Å.^56−59^ Dynamic contacts between 10 and 90% of each simulation were selected to evaluate the probability of contact formation (*p*_c_). The contact probability differences (d*p*_c_), from the wild-type to I335H variant were calculated to indicate the more often formed contact (d*p*_c_ ≥ 0.1) and less often formed contact (d*p*_c_ ≤ −0.1) probability differences. In difference contact network analysis (dCNA),^60^ contacts with a high probability of formation (*p*_c_ ≥ 0.9) in both wild-type and I335H variant simulations were selected to generate a consensus contact network. Communities were determined via Girvan–Newman’s algorithm^61^ on the consensus contact network and network modularity analysis.^62^ Community contact probability differences were defined as the net d*p*_c_ of residue–residue contacts between communities. Graphics of the analysis were obtained using Bio3D^63,64^ and igraph^65^ R packages.

### Site-Directed Mutagenesis, Protein Expression, and Purification of I335H

The plasmid pET20b(+)/PA3863 harboring the wild-type gene (*dau*A) encoding for d-arginine dehydrogenase was used as a template for site-directed mutagenesis. The PCR was carried out in the presence of 5% dimethyl sulfoxide in the reaction mixture to facilitate the denaturation of the GC-rich portions of the double-stranded DNA. The PCR product was purified using the QIA quick PCR purification kits and treated with *Dpn*I at 37 °C for 2 h. The resulting product was used to transform DH5α *E. coli* cells. The plasmid, pET20b(+)/PA3863/I335H, was sequenced at the DNA Core Facility of Georgia State University using an Applied Biosystems Big Dye Kit on an Applied Biosystems model ABI 377DNA sequencer to confirm the presence of the desired mutation. The pET20b(+)/PA3863/I335H harboring the mutation was used to transform the *E. coli* strain Rosetta(DE3)pLysS, which was stored at −80 °C. The I335H enzyme was expressed from Rosetta(DE3)pLysS cells and purified to homogeneity in the presence of 10% (v/v) glycerol via ammonium sulfate fractionation and ion exchange chromatography using the published procedure for the wild-type enzyme.^30^

### Steady-State Kinetics

The steady-state kinetic parameters with d-arginine or d-leucine as substrates for the I335H enzyme were determined using the method of initial rates using a Clark-type oxygen electrode (Hansatech Instrument).^30,66^ PMS was used as an electron acceptor since *Pa*DADH is a true dehydrogenase and does not react with molecular oxygen. The oxygen consumption in the reaction chamber is correlated with the enzymatic activity since the reduced PMS by the enzyme reacts spontaneously with molecular oxygen. The measurements were carried out in 20 mM sodium phosphate in the pH range from 5.5 to 10.0 at 25 °C with a fixed saturating concentration of 1 mM PMS. In the 1 mL reaction mixture, the enzyme concentration ranged from 0.05 to 15 μM and the substrate concentration ranged from 10 μM to 200 mM depending on the pH used. Ratios of [*S*]/[*E*] 100 ≥ 1 were used, and care was exerted to ensure that the *K*_m_ values, and consequently the *k*_cat_ and *k*_cat_/*K*_m_ values, were within the range of substrate concentrations used.

### Reductive Half Reaction

The reductive half reaction (RHR) of the I335H variant enzyme was performed aerobically under pseudo-first-order conditions in a stopped-flow spectrophotometer, SF-61DX2 Hi-Tech KinetAsyst, thermostated at 25 °C. Flavin reduction was monitored by following the absorbance changes at 445 nm in 20 mM sodium pyrophosphate or sodium phosphate, depending on the pH. d-Leucine was used instead of d-arginine as a reducing substrate because with d-arginine, more than 50% of flavin reduction occurred in the mixing time (i.e., 2.2 ms) of the stopped-flow spectrophotometer. A similar pattern was also observed in the wild-type and the Y53, Y249, S45, and A46 variants of *Pa*DADH, where flavin reduction occurred almost completely in the mixing time of the stopped-flow device.^31,42,67,68^ The RHR was investigated in the pH range 7.5–10.0 by mixing equal volumes of the enzyme and varying concentrations of the substrate from 0.1 to 15 mM. The enzyme was first gel filtered in a desalting PD-10 column to remove any unbound flavin; the final concentration of the enzyme-bound flavin after mixing was ∼10 μM. The kinetic isotope effects were determined with the deuterated substrate, d-leucine-*d*_10_,_,_ and by following the same procedures as that described above with the deuterated substrate. The absorption changes were collected in triplicate for each concentration of substrates used, and the average value was reported. Multiple measurements are typically different by ≤5%.

### Data Analysis

The data from the kinetic studies were fit using the KaleidaGraph software (Synergy Software, Reading, PA) and the Kinetic Studio Software Suite Enzfitter (Hi-Tech Scientific, Bradford on Avon, UK). The Michaelis–Menten equation for a single substrate was used to determine the apparent steady-state kinetic parameters at varying concentrations of the amino acid substrates.

Time-resolved flavin reduction data were fit to eq 1, which describes a single exponential process for flavin reduction. In eq 1, *k*_obs_ represents the observed first-order rate constant for the change in the absorbance at 445 nm associated with flavin reduction at any given concentration of the substrate, *t* is the time, *A* is the absorbance at 445 nm at any given time, *B* is the amplitude of the absorption changes, and *C* is the absorbance at infinite time of the fully reduced enzyme-bound flavin accounting for the non-zero absorbance.

The rate constant of flavin reduction (*k*_red_) was determined by fitting the *k*_obs_ values determined in the RHR to eq 2, where *k*_obs_ represents the observed first-order rate constant for the reduced enzyme-bound flavin at any given concentration of the substrate (*S*), *k*_red_ is the limiting first-order rate constant for flavin reduction at saturation concentrations of the substrate, *K*_d_ is the equilibrium constant defining the dissociation of the enzyme–substrate (ES) complex into the free substrate and enzyme, and *k*_rev_ is the limiting first-order rate constant of the reverse step in flavin reduction. *k*_rev_ in eq 2 corresponds to a finite y-intercept value that is not significantly different from zero.

The pH profiles of the *k*_cat_/*K*_m_, *k*_cat_, *k*_red_/*K*_d_, and *k*_red_ values with d-leucine as the substrate were fit to eq 3, which describes a profile that increases with increasing pH with a slope of +1, defining a single p*K*_a_ value and a pH-independent limiting value (*C*) at high pH. The pH profile on *k*_cat_ with d-arginine as the substrate was also fit to eq 4.

The pH profile of the *k*_cat_/*K*_m_ values with d-arginine as the substrate was fit to eq 4, which describes a profile that increases with increasing pH with a slope of +2, defining two indistinguishable p*K*_a_ values and a pH-independent limiting value (*C*) at high pH.
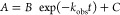
1
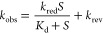
2
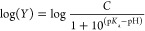
3
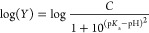
4

## Results

### Molecular Dynamics

**Figure 2:**
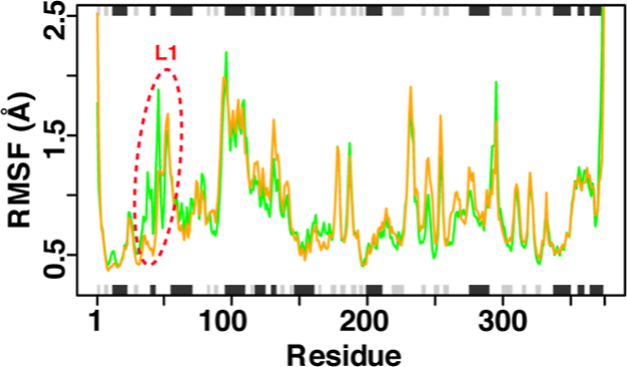
RMSF analysis showing backbone conformational dynamics. Residue-wise averaged RMSF of the wild-type (green) and I335H variant (orange) are calculated based on the backbone atoms. The black and gray bars at the top and bottom of the plot correspond to the α-helices and β-strands, respectively. Loop L1 (residues 33–56) is shown on the plot.

**Figure 3:**
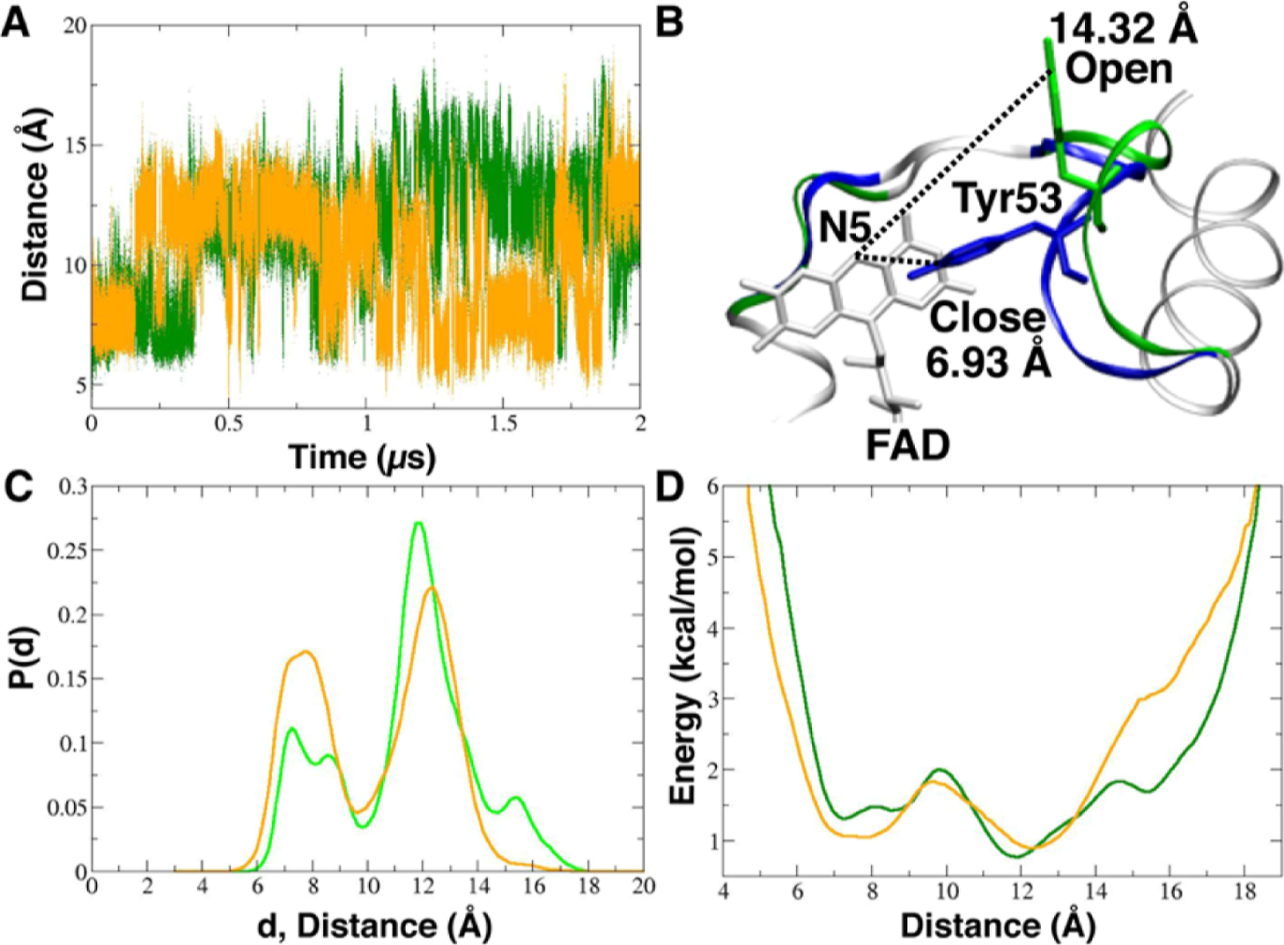
Distance analyses between Y53 (C4) and FAD (N5). (A) Distance over the simulation time frame between the C4 atom of Y53 and the N5 atom of FAD in the wild-type (green) and I335H variant (orange) trajectories. (B) Structural representation of the distance in the opened and closed conformations of PaDADH. (C,D) Probability distributions and free energy plots, respectively, of the distance in the wild-type (green) and I335H variant (orange).

The molecular dynamics simulations were carried out on both the I335H variant and the wild-type enzyme to investigate the impact of the mutation on *Pa*DADH. The RMSF studies of the I335H variant and the wild-type enzyme show similar backbone atomic fluctuations throughout the structure of *Pa*DADH except for the amino acid peptidyl region comprising residues 33–56, which in previous studies^42,43^ of the enzyme was denoted as loop L1 (Figure 2). This loop adopted an open and closed conformational state in which Y53 acts as a gate to control substrate accessibility and product release from the active site. In contrast, the amino acid peptidyl region of loop L4 comprising residues 329–336, which contains I335, has minor atomic backbone fluctuations (Figure 2). The distance analysis showed the impact of the mutation on the open and closed conformations of the Y53 gate on both the I335H variant and the wild-type enzyme (Figure 3). Previous root mean square deviation (rmsd) analyses^42^ on the FAD cofactor showed a localized flavin component, not only in the wild-type enzyme but also in the mutant variants of interest. The rmsd fluctuation of FAD remained relatively stable throughout the simulations; thus, the distance analyses between the N5 atom of FAD and the C4 atom of Y53 were able to probe the open and closed state of the enzyme. The FAD cofactor is embedded in the FAD binding domain^43^ of the enzyme, which restricts changes in its dynamics in both the wild-type and the I335H enzyme variant. The time courses of the distance between the C4 atom of Y53 and the flavin N5 atom show that both enzymes rapidly sample many conformational states over the simulation time frame but with different probabilities of being in the open and closed conformations (Figure 3A–C). The I335H variant displays higher probability distributions in closed and less populated open conformations compared to the wild-type enzyme (Figure 3C). This ultimately translates in the free energy plots (Figure 3D) by showing a lower barrier from the open conformation to closed conformation in the I335H variant than in the wild-type enzyme.

**Figure 4:**
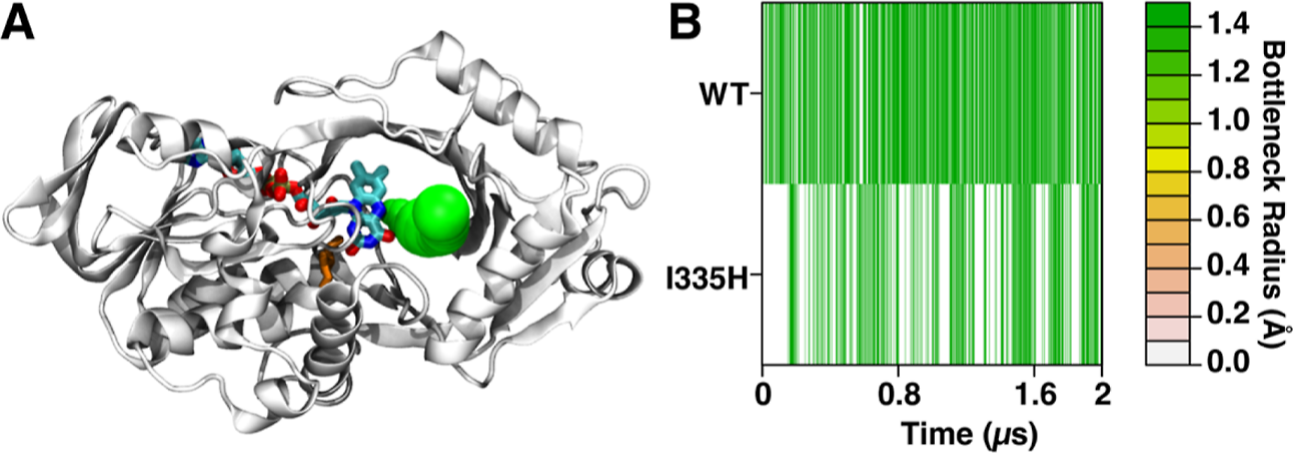
Analyses of the substrate entrance cavity in *Pa*DADH. (A) Snapshot model of the substrate entrance cavity (green) using the N5 atom of the FAD as the point of origin. I335 (orange) is shown at the *si* face of the flavin. The FAD is shown as licorice. (B) Bottleneck radii over the simulation time frame of the wild-type and the I335H variant. Bottleneck radii are shown from narrow (white) to wide (green) measurements.

A CAVER analysis was conducted to probe the change of the active site cavity of *Pa*DADH over the simulation time frame. Figure 4A represents a snapshot of a cavity formed once the enzyme is in the open conformation. Cavities formed throughout the trajectories of the I335H variant and the wild-type enzyme were studied based on their bottleneck radii to generate a heat map of this dimension over the simulation time frame for both systems. Interestingly, due to the active site lid (i.e., loop L1) and the gating properties of Y53, the active site cavity can either be fully shielded or opened to the bulk solvent but not be partially open (Figure 4B). The bottleneck radii in the CAVER analysis show cavity openings more frequently over the simulation time frame in the wild-type than in the I335H variant.

**Figure 5:**
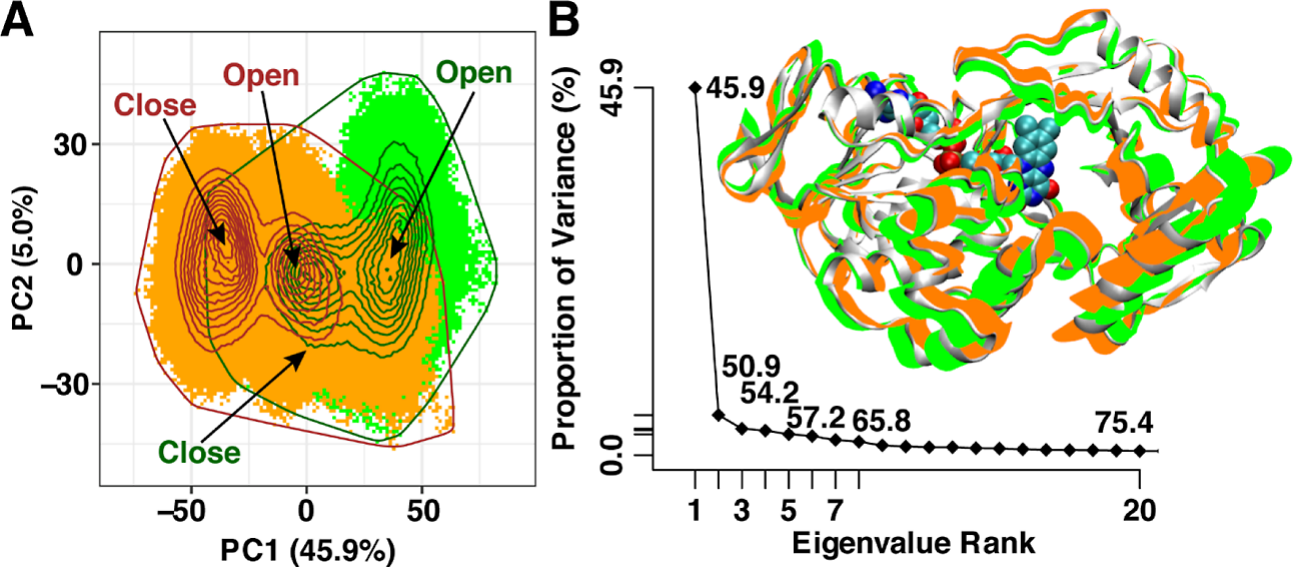
PCA on the wild-type and I335H variant. (A) PC1 and PC2 plots of the wild-type and I335H trajectories. The total atomic displacements/variance captured by PC1 and PC2 are shown in the axis labels. Wild-type (green) and I335H (orange) outlines are shown to indicate the subspace spanned by both trajectories. The probability density distributions of the conformational states are shown as contour lines. The open and closed conformations of the wild-type (green) and I335H (orange) are shown on the PCA plot. (B) Scree plot indicating the eigenvalue spectrum and the collective PC1 motions of the wild-type (green) and I335H (orange) projected onto PaDADH with the FAD shown as VDW.

The PCA showed two distinct conformational spaces for the I335H variant and the wild-type enzyme along PC1 (Figure 5A), which indicates the open and closed conformational states in both enzymes. As shown in Figure 5A, the PCA is consistent with the I335H variant sampling more in the closed conformation than the wild-type enzyme. Projection of PC1 and PC2 onto *Pa*DADH (Figure 5B) shows a clear variation in the overall conformational change sampled in the wild-type enzyme (green) compared to that in the I335H variant (orange). As shown in the eigenvalue spectrum (scree plot; Figure 5B), PC1 and PC2 account for more than 50% of the total structural variance, which suggests that *Pa*DADH is mainly sampling two conformational states.

**Figure 6:**
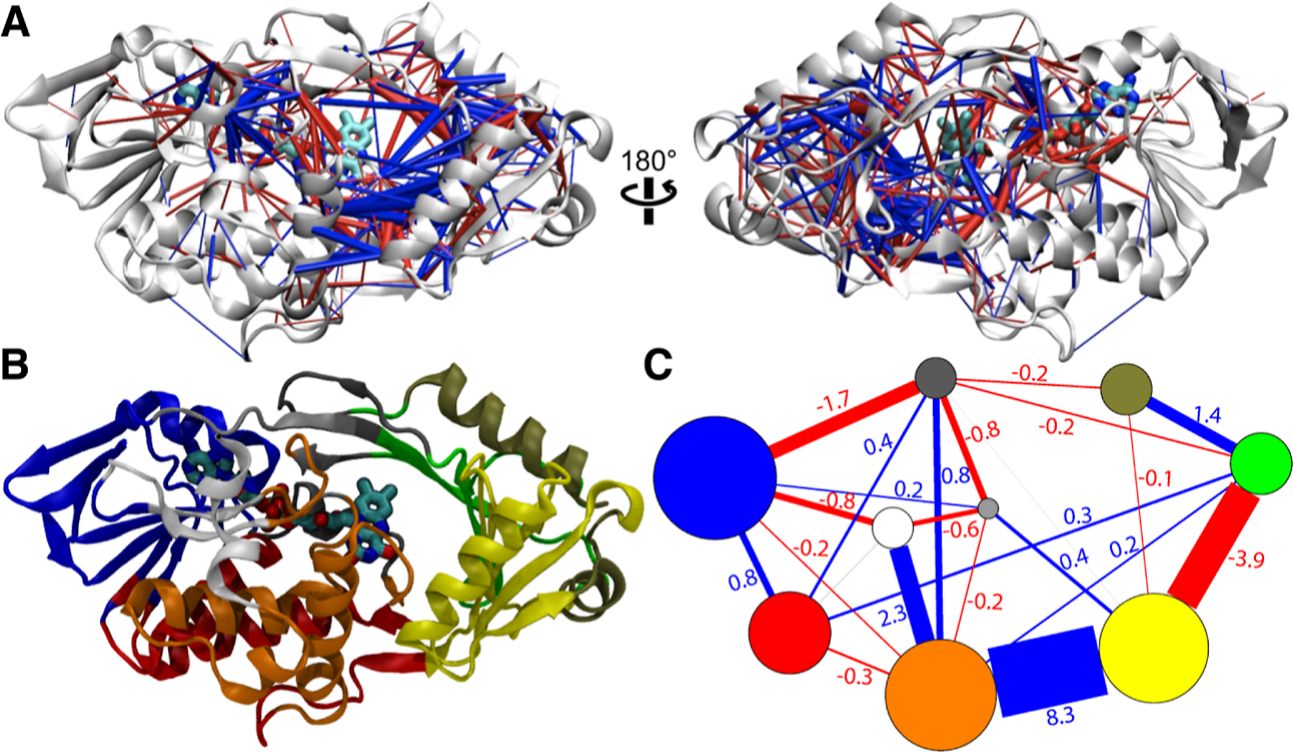
Contact statistics from the wild-type to the I335H variant. (A) Residue–residue contact probability differences (d*p*_c_) from the wild-type to the I335H variant. Contacts with a higher formed probability (d*p*_c_ ≥ 0.1) are shown as blue cylinders and less often formed contacts (d*p*_c_ ≤ −0.1) are shown as red cylinders. (B) Structural representation of PaDADH in dCNA with nine communities of different colors. FAD is drawn in licorice within the FAD-binding pocket of the enzyme structure. (C) Community networks of *Pa*DADH from the wild-type to the I335H variant. The radii of vertices correspond to the number of residues within the communities. Communities in (B) have the same color as vertices in (C). Net residue–residue contact probability differences are shown as red and blue lines, with red representing net contacts less often formed and blue showing net contacts more often formed between the communities.

The residue–residue contact probability differences (d*p*_c_), as shown in Figure 6A, with a 90° rotation of the structure indicate that there are more contacts formed in the substrate-binding domain (blue cylinder lines) than in the FAD-binding domain. The dCNA data show a total of nine consensus communities (Figure 6B,C) spanning both the FAD and the substrate-binding domains. The FAD-binding domain contains six communities colored in blue, white, red, black, gray, and orange, which harbors the I335 residue. The substrate-binding domain contains three communities colored green, tan, and yellow (Figure 6B,C). The community contact networks from the wild-type to the I335H variant, as shown in Figure 6C, indicate the presence of strong interactions (and hence domain closure) that are being formed between the communities upon the I335 mutation.

### Purification of the I335H Variant

*Pa*DADH I335H was expressed and purified to high levels using the same protocol as that described previously for the wild-type enzyme.^41^ Since the cofactor is non-covalently bound to the enzyme, 10% glycerol (v/v) was added in each step of the purification to increase the enzyme stability and prevent the possible loss of flavin.

### pH Effect on the Steady-State Kinetic Parameters with d-Arginine and d-Leucine

**Figure 7:**
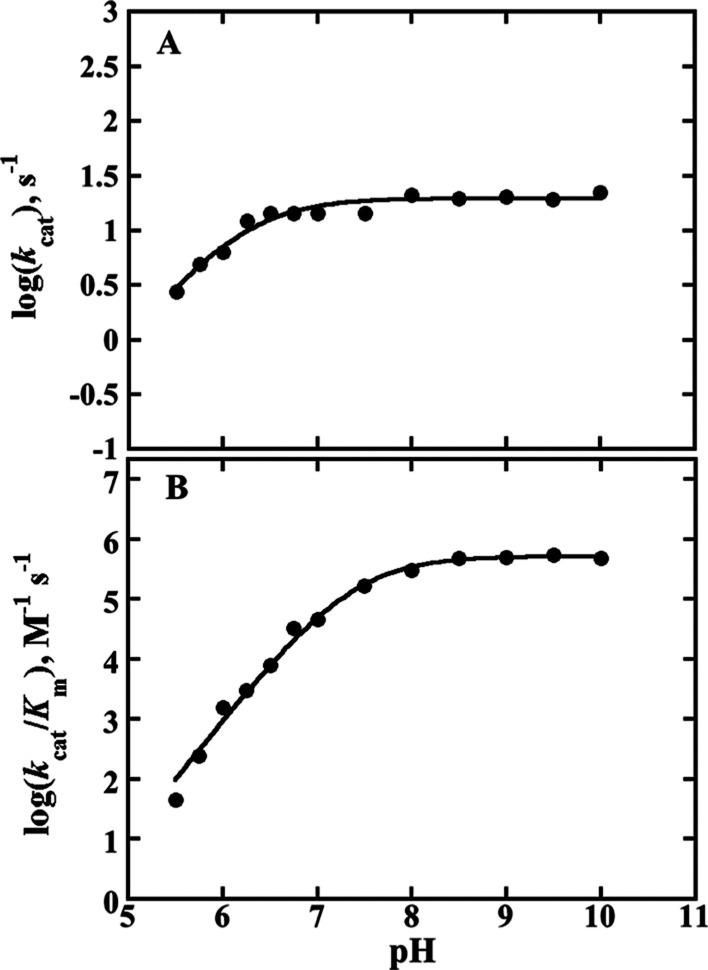
pH profiles of *k*_cat_ (A) and *k*_cat_/*K*_m_ (B) with d-arginine as the substrate. The I335H variant activity was measured at varying concentrations of d-arginine at a fixed saturation concentration of 1 mM PMS at 25 °C as described in the experimental procedures. The lines in panel A and B are fits of the data, respectively, to eqs 3 and 4.

**Figure 8:**
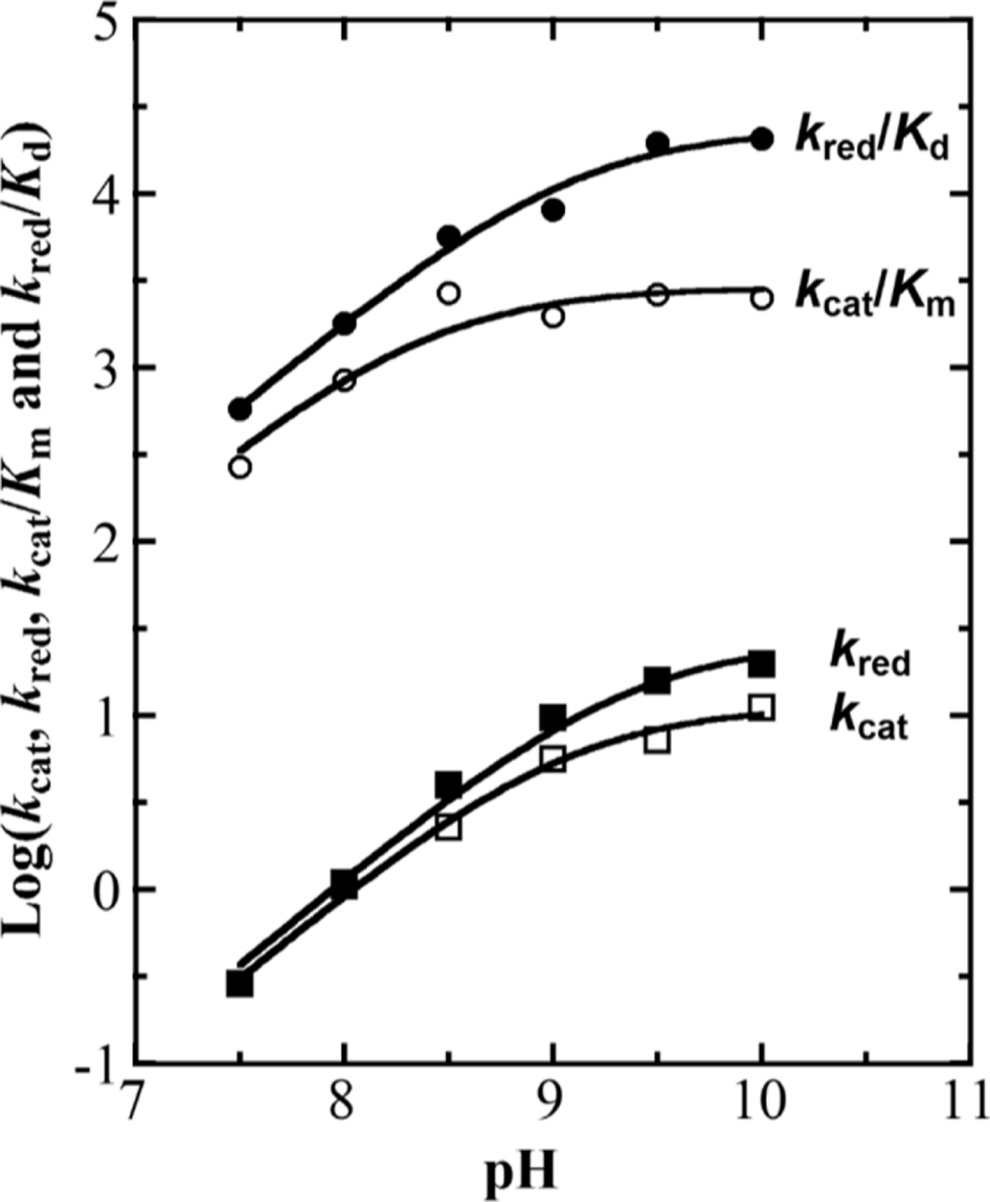
pH dependence of the I335H variant with d-leucine as the substrate. The kinetic parameters *k*_cat_ (□), *k*_red_ (■), *k*_cat_/*K*_m_ (○), and *k*_red_/*K*_d_ (●) values of the I335H variant with d-leucine as the substrate were measured at varying concentrations of d-leucine and 1 mM PMS at 25 °C. The kinetic parameter data were fit to eq 3.

The pH effects on *k*_cat_ and *k*_cat_/*K*_m_ with d-arginine and d-leucine were characterized to investigate the impact of the mutation on any ionizable group on both substrate capture and catalysis. The log(*k*_cat_) with d-arginine (Figure 7A) and d-leucine (Figure 8) as well as the log(*k*_cat_/*K*_m_) with d-leucine (Figure 8) all increased with increasing pH values, reaching limiting values at high pH and displaying the requirement of a single unprotonated group. However, the log(*k*_cat_/*K*_m_) with d-arginine (Figure 7B) increased with a slope of +2 to a limiting value at high pH, defining the requirement of two unprotonated groups. The p*K*_a_ and the pH-independent values on the kinetic parameters *k*_cat_ and *k*_cat_/*K*_m_ with d-arginine and d-leucine as substrates for both enzymes are summarized in Tables 1 and 2.

**Table 1:** pH-Independent and p*K*_a_ Values of I335H with d-Arginine as the Substrate^c^

	pH-independent values				
	*k*_cat_/*K*_m_			*k*_cat_	
enzymes	p*K*_a1_	p*K*_a2_	(*k*_cat_/*K*_m_)_H_^a^ (M^–1^ s^–1^)	p*K*_a1_	(*k*_cat_)_H_^a^ (s^–1^)
I335H	7.4 ± 0.1	7.4 ± 0.1	500,000 ± 77,000	6.2 ± 0.1	20 ± 1
wild-type^b^	7.2 ± 0.1	7.2 ± 0.1	2,600,000 ± 500,000	6.3 ± 0.1	160 ± 20

a pH-independent limiting number determined at high pH.

b Wild-type data were taken from ref (68).

c *k*_cat_/*K*_m_ and *k*_cat_ values were determined, respectively, by using eqs 3 and 4.

**Table 2:** pH-Independent and p*K*_a_ Values of I335H with d-Leucine as the Substrate

	pH-independent values			
enzyme	*k*_red_ (s^–1^)	*k*_red_/*K*_d_ (M^–1^ s^–1^)	*k*_cat_ (s^–1^)	*k*_cat_/*K*_m_ (M^–1^ s^–1^)
I335H	32 ± 6	24,000 ± 3000	11 ± 1	2900 ± 400
wild-type^a^	133 ± 5	12,000 ± 1200	100 ± 15	11,000 ± 1500
	p*K*_a_ values			
I335H	9.5 ± 0.1	9.1 ± 0.1	9.1 ± 0.1	8.4 ± 0.1
wild-type^a^	9.6 ± 0.1	9.1 ± 0.1	9.5 ± 0.1	9.5 ± 0.1

a Data for the wild-type *Pa*DADH are from ref (68).

### pH Effects on the RHR with d-Leucine

**Figure 9:**
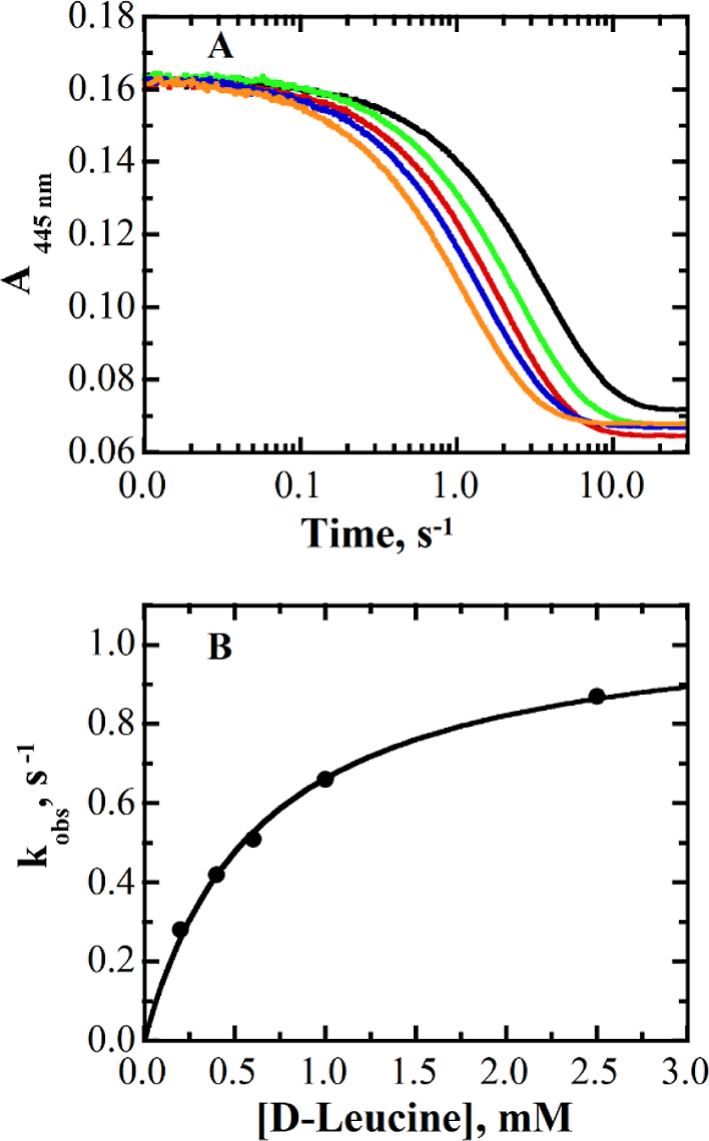
RHR of the I335H variant with d-leucine as the reducing substrate.

The pH effects on the RHR of the I335H variant were investigated to evaluate the effects of the mutation on the different kinetic parameters in the pH-independent region and to use those values with the steady-state kinetic parameters to compute the kinetic rate constants of I335H in the RHR. As previously established for wild-type *Pa*DADH, the RHR with d-arginine as a substrate for the I335H enzyme could not be studied because most of the reduction of the enzyme-bound flavin occurred within the mixing time of the stopped-flow spectrophotometer.^31^ Thus, the alternate, slow substrate d-leucine was used instead of d-arginine as the reducing substrate to gain insights into the rate constant associated with the RHR in the I335H enzyme. Flavin reduction was monitored by following the absorbance changes at 445 nm under pseudo-first-order conditions upon mixing the enzyme with d-leucine in a stopped-flow spectrophotometer. As illustrated in Figure 9 for the case of pH 8.0, the stopped-flow traces were best fit to a single exponential process, indicating a hyperbolic dependence of the observed rate constant for flavin reduction (*k*_obs_) on the concentration of d-leucine.

The *k*_red_, *K*_d_, and *k*_red_/*K*_d_ values could be determined between pH values of 7.5 and 10.0 (Figure 8). Both the *k*_red_ and *k*_red_/*K*_d_ values increased with increasing pH, reaching plateaus at high pH, as previously established for wild-type *Pa*DADH. The pH-independent value for *k*_red_ was 4 times lower, whereas the *k*_red_/*K*_d_ value was 1.7 times larger in the I335H enzyme than in wild-type *Pa*DADH (Table 2). In contrast, the p*K*_a_ values determined for *k*_red_ and *k*_red_/*K*_d_ were not significantly different between the I335H variant and the wild-type enzyme (Table 2). The *K*_d_ value in the I335H enzyme was independent of the pH with an average value of 0.8 ± 0.3 mM (data not shown). For comparison, the wild-type enzyme has a *K*_d_ value of 3.5 ± 0.3 mM between pH values of 7.0 and 9.5.^31^

The experiment was carried out in phosphate buffer at a pH of 8.0 and 25 °C. Panel A: stopped-flow traces of the absorption changes at 445 nm when the enzyme is mixed with 0.2 mM (black), 0.4 mM (green), 0.6 mM (red), 1.0 mM (blue), and 2.5 mM (orange) d-leucine. All traces were fit to eq 1. Panel B: dependence of the observed rate constant for flavin reduction on the concentration of d-leucine, with data fit to eq 3.

## Discussion

The kinetic and computational data are consistent with I335 being part of a residue network that controls the overall conformational dynamics of *Pa*DADH. The RMSF data show that loop L4, which harbors I335 and comprises residues 329–336, fluctuates with a similar pattern in both the I335H variant and wild-type enzyme (Figure 2). Loop L4 is buried in the active site pocket and spans around the ribityl moiety of FAD and the hydrophilic portion of the isoalloxazine ring of FAD (Figure 1). Furthermore, the RMSF data are consistent with the peptidyl region of loop L1 being the most flexible region in the protein. As shown in Figure 1, I335 in loop L4 is located on the opposite side of the substrate-binding pocket and does not interact directly with the iminoarginine product. In the closed conformation, only Q336 in loop L4 has a polar interaction with the backbone nitrogen and oxygen atoms of T50 in loop L1, while there is a hydrogen bond interaction between T50 and the guanidinium group of the iminoarginine product of the enzyme–product (EP) complex.

The replacement of I335 with histidine in loop L4 shifts the conformation dynamics of the enzyme to the sample more in a closed conformation. Evidence in support of the mutation favoring a closed conformation comes from the distance and CAVER analysis studies, as shown in Figures 3 and 4, respectively. Recent work on *Pa*DADH showed that the dynamics of loop L1 are important for catalysis, where Y53 adopts open–closed conformations to allow substrate entrance and product exit from the active site (Figure 3B).^42^ Therefore, a distance distribution analysis (Figure 3A) was carried out to evaluate the impact of the I335H mutation on the open–closed conformation of the Y53 gate. In this study, a distance of ≤7 Å between the C4 atom of the Y53 gate and the N5 atom of the flavin is defined as the “closed” conformation, while a distance of ≥14 Å is considered as the “open” conformation. The open and closed conformation distances are derived from the crystal structures of the wild-type enzyme denoted as state A and B, respectively. As shown in the probability distribution plot (Figure 3C), the Y53 gate in the I335H variant samples more in the closed conformation than in the wild-type enzyme. This is consistent with the free energy plots (Figure 3D) showing a lower energy barrier from the open conformation to the closed conformation in the I335H variant than in the wild-type enzyme. The CAVER analysis compares the tunnel access to the active site cavity of the I335H variant relative to that of the wild-type enzyme (Figure 4A). The bottleneck radii in the I335H variant show cavity openings less frequently over the simulation time frame compared to that in the wild-type enzyme (Figure 4B). The CAVER analysis and the distance distribution data are consistent with the mutation changing the ensemble configuration of the active site residues toward a more packed and closed conformation.

Further evidence supporting a closed conformation due to the mutation at position 335 in loop L4 comes from the PCA data showing two distinctive conformational spaces corresponding to “open” or “closed” conformations along PC1 for both the I335H variant and the wild-type enzyme. As shown in Figure 5A, the open conformational states of the I335H variant correspond to the closed conformational states of the wild-type, which is consistent with the I335H variant sampling more in the closed conformation than the wild-type. Moreover, the overall conformation of *Pa*DADH is perturbed toward a closed conformation in the I335H variant, as demonstrated by the projection of both PC1 and PC2 in Figure 5B. Thus, I335 in loop L4 behaves like a “hot spot” in the residue network that controls the open and closed conformations of *Pa*DADH. More evidence to support this conclusion comes from the residue–residue contact probability differences (d*p*_c_) and the dCNA. The contact statistics from the wild-type enzyme to the I335H variant suggest the closure of the active site cavity from the net d*p*_c_ of 8.3 between the orange and yellow communities (Figure 6C), consistent with a long-range effect of a single point mutation on the overall dynamics of the enzyme. I335H mutation, located within the orange community, shows net d*p*_c_’s with more often formed contact and less often formed contact probabilities with surrounding communities. Indeed, the orange and yellow communities have a net d*p*_c_ of 8.3, while the yellow community shows a net d*p*_c_ of −3.9 with the green community. This community contact network ultimately hinders access to the substrate-binding pocket. The white community shows net d*p*_c_’s of −0.6, −0.8, and 2.3 with the gray, blue and orange communities, respectively, indicating a structural change in the FAD-binding domain from the mutation in the orange community. The residue and community contact analysis suggests a concerted impact of the mutation with surrounding residues, which propagates to different compartments of the protein. This propagation of interactions includes a combination of both electrostatic and steric effects.

**Scheme 2:**
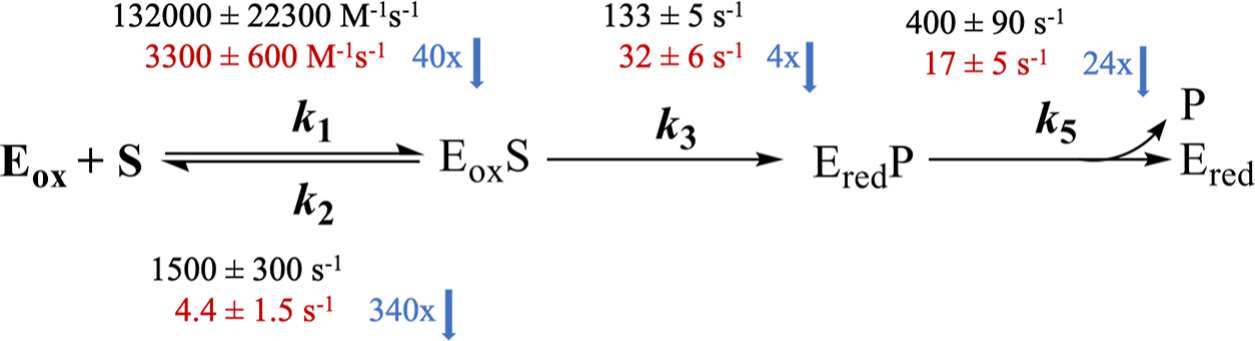
Microscopic Rate Constants of the I335H Variant (Red) Compared with That of the Wild-Type (Black)

The dynamic effects generated by the I335H mutation impact the catalytic function of the enzyme. Evidence to support this conclusion comes from the kinetic data, as shown in Scheme 2.

In fact, the mutation affects *k*_1_, the rate constant of the free enzyme to capture the free substrate to form the ES complex; *k*_2_, the rate constant for the dissociation of the ES complex to the free enzyme and free substrate; and *k*_5_, the rate constant for product release from the EP complex significantly (Scheme 2). The rate constant *k*_1_ was decreased by 40-fold, *k*_2_ was decreased by 340-fold, and *k*_5_ was decreased by 24-fold compared to that of the wild-type enzyme (Scheme 2). In contrast, *k*_3_, the rate constant of flavin reduction, was only decreased by 4-fold, which is consistent with the I335H mutation not significantly affecting the chemical step. Indeed, the microscopic rate constants *k*_1_, *k*_2_, and *k*_5_ report mainly on the ability of the enzyme to accommodate its ligands and are intrinsically linked to the conformational flexibility of the enzyme. The difference in both *k*_2_ and *k*_5_ values is mostly due to a different interaction between the two ligands with the active site residues due to the mutation. The evidence to support both the kinetic mechanism and the calculation of the microscopic kinetic rate constants in Scheme 2 is provided below.

Previous studies established that *Pa*DADH operates through a Ping–Pong Bi–Bi steady-state kinetic mechanism for catalysis, in which the oxidation of the reduced flavin by the artificial electron acceptor PMS occurs after the release of the imino acid product of the reaction.^30^ Furthermore, it was shown that the oxidative half reaction with PMS is fast, which indicates that the kinetic steps of the oxidative half reaction do not contribute to the overall catalytic turnover of the enzyme. Thus, the overall turnover in *Pa*DADH is mainly controlled by the kinetic steps of the RHR.

The rate constant of flavin reduction (i.e., *k*_3_ = *k*_red_) is irreversible in the I335H variant. As shown in Figure 9, the dependence of the observed rate constants for flavin reduction (*k*_obs_) on the substrate concentration was fit to eq 2, which describes a hyperbolic trend without a *y*-intercept (*k*_rev_). The absence of a y-intercept is consistent with the rate constant of flavin reduction in the reverse direction being negligible (i.e., *k*_4_ = *k*_rev_ ∼ 0). A previous study showed that the rate of flavin reduction is irreversible in the wild-type enzyme with d-leucine as a substrate irrespective of the pH being between 7.0 and 11.^31^ However, studies on the Y53F and Y249F variants of *Pa*DADH showed that the rate of flavin reduction in these tyrosine mutant variants was reversible.^67^

The rate constant for flavin reduction *k*_red_ is partially rate-limiting in the I335H variant. Evidence supporting this conclusion comes from the measured *k*_red_ value of 32 ± 6 s^–1^ compared to the overall turnover number, *k*_cat_, of 11 ± 1 s^–1^, consistent with another kinetic step besides *k*_red_ contributing to the overall turnover. Since the catalytic cycle of *Pa*DADH operates through a Ping–Pong Bi–Bi kinetic mechanism in the steady state and the oxidative half reaction with PMS as an electron acceptor is fast,^30^ the only first-order kinetic step beside the rate of flavin reduction that is partially rate-limiting in the RHR is the rate of product release, *k*_5_ (Scheme 2).

The overall turnover number (*k*_cat_) is contributed mainly by the rate of flavin reduction and product release. This conclusion is supported by the derived expression of *k*_cat_ in the I335H variant, as shown in eq 5, where *k*_3_ represents the rate of flavin reduction (*k*_red_), which is experimentally measured. The rate constant for product release, *k*_5_, of the I335H variant, was calculated using eq 5 in which both *k*_3_ and *k*_cat_ values are known (Table 2). The value of *k*_5_ for the I335H variant was 17 ± 5 s^–1^ compared to 400 ± 90 s^–1^ in the wild-type. The rate constant *k*_1_ was calculated from both the expressions of *k*_cat_/*K*_m_ and *k*_red_/*K*_d_ in eqs 6 and 7, respectively.^69^
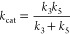
5
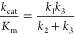
6
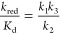
7

The *k*_cat_/*K*_m_ and *k*_red_/*K*_d_ values determined with d-leucine differ in their values, indicating that their analytical expressions are also different. The simplest explanation to reconcile these data without invoking additional kinetic steps for which there is no experimental evidence is that the analytical expression for *k*_red_/*K*_d_ is given by eq 7. By taking the reciprocal of the *k*_cat_/*K*_m_ expression (eq 6) and substituting the term *k*_2_/(*k*_1_*k*_3_) with the reciprocal of eq 7, one obtains eq 8, which can be used to calculate *k*_1_. The calculated value of *k*_1_ in the I335H variant was 3300 ± 600 M^–1^ s^–1^ compared to 132,000 ± 22,300 M^–1^ s^–1^ in the wild-type. The rate constant for the dissociation of the ES complex to the free enzyme and free substrate, *k*_2_, is then computed from eq 7 by using the *k*_red_/*K*_d_ and *k*_3_ values experimentally determined and the computed *k*_1_ value. The computed value of *k*_2_ was 4.4 ± 1.5 s^–1^ in the I335H variant compared to 1500 ± 300 s^–1^ in the wild-type.
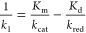
8

## Conclusions

In conclusion, we have used both computational and experimental techniques to investigate the role of the I335 residue in *Pa*DADH. I335 is located in a rigid and buried loop L4 and positioned at least 10 Å away from the active site and close to the N(1)–C(2)=O region of the flavin cofactor. The kinetic and computational data are consistent with I335 being part of a residue network with dynamically coupled long-range effects that control both the dynamics and catalytic function of *Pa*DADH. The molecular dynamics studies, including distance distribution studies of the Y53 gate, CAVER, PCA, and dCNA, demonstrated a shift in the conformational dynamics to a more closed conformation in the I335H variant enzyme. Thus, the change of the conformational dynamics due to the mutation significantly perturbed the accommodation of both the ES and EP complexes in *Pa*DADH. The kinetic data in the I335H variant showed a decrease of 40-fold in *k*_1_, a decrease of 340-fold in *k*_2_, and a 24-fold decrease in *k*_5_, the rate of product release. However, a lesser impact was observed on the chemical step *k*_3_, which was lowered by only 4-fold. The data are consistent with the mutation preventing access and exit of ligands from the active site, resulting in the accumulation of the ES and EP complexes. This study shows the significance of the synergistic effects of residues outside the active site pocket that could affect catalysis through long-range effects, as demonstrated by both computational and kinetic studies.

## Notes

UniProt ID: Q9HXE3: d-arginine dehydrogenase.
